# Celluloid

**Published:** 1876-01

**Authors:** C. C. Burt

**Affiliations:** late Solicitor for the Dentists.


### SELECTED ARTICLES.

### ARTICLE III.

### *Celluloid.*

JACKSON, MICHIGAN, SEPT. 22ND, 1875.

*Mr. Editor:—*

In looking over the September number of your well written *Journal*, I noticed an allusion to the prosecution for the use of celluloid in dentistry, and a hint as to its infringement of the John A. Cummings patent, of 1864. This calls to mind a circular shown to me by Dr. J. A. Robinson of this city, in which *Josiah* threatens that he will prosecute any dentist who shall have the temerity to refuse to take a hard rubber license, and use in lieu thereof celluloid, or that its use as a base or otherwise in artificial dentures is an infringement of the Cummings patent. At that time I paid very little attention to it, considering it some of the vaporising effusiveness of Josiah uttered while smarting under the castigation he and the Cummings' patent received in the Willis case at Detroit, when he undertook to force the Michigan dentists to pay royalty for the use of his pretended Cummings' patent. I paid very little attention to it; but since that time having been frequently inquired of by dentists, both in writing and in person, whether the use of celluloid was an infringement of that patent, my invariable answer has been *no*, without giving the reason for my belief, and knowing that many dentists were using this substance instead of rubber, the article in your September number prompted me to write this article, which, should you deem of sufficient importance, you are at liberty to print. My reasons for saying that celluloid use is no infringement of the Cummings' patent is two-fold:

First.—Because it is neither rubber, vulcanite, gutta percha or allied gums; it cannot be compounded with sulphur or iodine, not vulcanized, neither is it a soft plastic substance hardened by vulcanization—Cummings' patent is for the use of "rubber vulcanite or allied gums, mixed with sulphur or like substances and vulcanized by the process of Goodyear." The allied gums are those exuding from tropical shrubs or trees, differing only from each other in soil and climate, like the various gums of the fir trees, that yield in some climates balsam or tar, while in colder climates and different soil they exude pitch and rosin. The allied gums contain all the necessary ingredients for use, while celluloid is collodion, gun cotton and camphor, rendered dense by a high process of condensation, and is now vulcanizable. In the argument of the Willis' case before the full bench of judges in the United States Court last November, speaking of this Cummings' patent,—see Burt's argument printed on the history of Dental Legislation in America, page 804, &c.—I said, "If your honors please, among other things I contend that this patent is void for uncertainty, and for covering too much ground. Pray what are allied gums? as claimed in the re-issue of this extraordinary fraud, called a patent. Can it be possible that our patent laws go to the romantic extent of foreclosing all and every device or product that the ingenuity of man may seek out, for each and every compound that this man Bacon shall claim to be "an allied gum." Under this, to say the least, very ambiguous and very general word, allied gums, if such is the law, and this most extraordinary worded claim can be sustained without doing violence to the well established rulings and precedents, the next thing your Honors will be called upon to enforce, will be punishment as infringers for these dentists who use celluloid for dental purposes."

By the court, Judge Emmons interrupting, "I hardly think, Bro. Burt, that any well posted patent lawyer would take such a position. I understand that the process, compound and product, is entirely dissimilar, how is that Bro. Lee? (Mr. Bacon's solicitor,) Mr. Lee—"If your Honors please, we do not, or ever claim the use of celluloid to be an infringement of our patent, it is not an allied gum, it is not a natural, but an artificial gum, and Bro. Burt need give himself no trouble on that score. *We claim only those natural Gums* that are, or can be, vulcanized by Goodyear's process, or in the language of our patent, a soft and plastic substance of rubber or allied gum, that is hardened by vulcanization, which celluloid is not."

So you see, that before full bench of judges, who were willing to go (as we think) a great way to sustain this, in my judgment, most infamous swindle, the Cummings' patent were *startled* at the idea of extending it to celluloid--and Bacon, by the most solemn and binding act, before that high tribunal, conceded that the use of celluloid *was no* infringment of the Cummings' patent; but now when he finds that celluloid is a dangerous rival, that the dentist who formerly paid him royalty, declined to buy his patent or use rubber, and that by his dishonest course of litigation, he has killed the bird that laid the golden egg, we must expect from this mercurial and volatile little fellow, some ebulitions of wrath; so let him roar; there may be wind, noise, and dust, but no shower; he has too much good sense to begin *such* a fight, and, then too when the celluloid company are ready to back up the refractory dentist who does disobey *Josiah's* mandate—it won't win. Bacon beat us with his everlasting patent, and the money wrung from the labor and genius of the dentists, verifying the old adage that every dog must have his day. But rubber and the Goodyear Horse Leach, spoken of in Holy writ; is dead; celluloid—killed—and poor Josiah is the principal mourner. So mote it be.

Since writing the above, which I had intended for the October number of your journal, I have received a printed circular from Josiah (which I presume he has sent to all the dentists) threatening direful punishment for infringment to those who use celluloid without a license, ridiculing Bro. Baldwin, and charging him with ruining the dentists, and which seems to call for a word of caution to all dentists. Remember how he beat us in the rubber fight, by obtaining by negligence or connivance several decrees, which were either neglected or poorly defended, and then claimed as real bona fide cases, and the cry of "re-adjudicate" set up in bar of our proceedings. He does not sue any one in Michigan, and I hope this warning will reach the dentists of other states; if sued, send at once your papers to the Celluloid Company, who I understand will defend all who use their manufactures. Don't let him get an inch the start and you have nothing to fear. Respectfully, &c.,

C. C. Burt, late Solicitor for the Dentists.

—*Missouri Dental Journal.*

---

